# Advances in Understanding the Effects of Erythropoietin on Renal Fibrosis

**DOI:** 10.3389/fmed.2020.00047

**Published:** 2020-02-21

**Authors:** Yangyang Zhang, Xiaoyu Zhu, Xiu Huang, Xuejiao Wei, Dan Zhao, Lili Jiang, Xiaoxia Zhao, Yujun Du

**Affiliations:** Department of Nephrology, The First Hospital of Jilin University, Changchun, China

**Keywords:** chronic kidney disease, renal fibrosis, erythropoietin, extracellular matrix, myofibroblasts

## Abstract

Renal fibrosis is the common manifestation of the pathogenesis of end-stage renal disease that results from different types of renal insult, and is a hallmark of chronic kidney disease (CKD). The main pathologic characteristics of renal fibrosis are renal interstitial fibroblast hyperplasia and the aberrant and excessive deposition of extracellular matrix, pathologies that lead to the destruction of normal renal tubules and interstitial structures. However, the biological significance of fibrosis during the progression of CKD is not clear, and there are no approved clinical treatments for delaying or reversing renal fibrosis. Studies of the mechanism of renal fibrosis and of potential measures of prevention and treatment have focused on erythropoietin (EPO), a hormone best known as a regulator of red blood cell production. These recent studies have found that EPO may also provide efficient protection against renal fibrosis. Future therapeutic approaches using EPO offer new hope for patients with CKD. The aim of the present review is to briefly discuss the role of EPO in renal fibrosis, to identify its possible mechanisms in preventing renal fibrosis, and to provide novel ideas for the use of EPO in future treatments of renal fibrosis.

## Introduction

The prevalence of chronic kidney disease (CKD) has risen significantly during the last several decades, and it is now a major public health issue that poses enormous economic challenges worldwide (1). According to recent epidemiological surveys, more than 500 million people worldwide currently have some stage of CKD (2, 3). The two principal causes of CKD are diabetes and hypertension; several additional conditions often accompany CKD, such as HIV infection, obesity, and primary kidney injury (4). The progression of CKD is accompanied by multiple complications and comorbidities that ultimately lead to renal fibrosis and end-stage renal disease, a condition associated with poor outcome and increased risk of death (5, 6).

Renal fibrosis is a progressive pathophysiological change that is associated with damage and functional loss of the kidney (7). Pathogenesis begins following stimulation by a variety of pathogenic factors, such as trauma, infection, inflammation, blood circulation disorders, and excessive immune responses. These insults damage healthy kidney cells, leading to the deposition and accumulation of large amounts of collagen as disease progresses. During the later stages of pathogenesis, there is a gradual hardening of the renal parenchyma and formation of scar tissue, and then the complete loss of kidney function (8). Renal fibrosis is simply the process of fibrosis and hardening of the formerly healthy cells of the kidney. It is also characterized by the abnormal deposition of extracellular matrix (ECM). Although previous studies have examined the main molecular mediators of renal fibrosis, there are no approved therapies that directly target renal fibrosis (9, 10). Therefore, it is necessary to further study the pathogenesis of renal fibrosis to facilitate the development of new drugs that inhibit renal fibrosis and delay the progression to CKD.

In 1906, Carnot and Deflander first proposed the existence of a hormone primarily produced by the kidneys that regulates red blood cell production. This hormone, subsequently named erythropoietin (EPO), is now known to regulate the formation of red blood cells by stimulating bone marrow, and is widely used for the clinical treatment of anemia caused by various factors (11–13). Recent research in China and elsewhere has examined the non-hematopoietic aspects of EPO (14–16). There is now evidence that EPO is a multifunctional cytokine (17). Thus, in addition to regulating erythropoiesis (which increases tissue oxygen supply) EPO also has anti-apoptosis, anti-inflammatory, antioxidant, angiogenesis, cell proliferation, and anti-tumor effects (18).

The aims of this review were to briefly review the scientific evidence regarding the relationship of the therapeutic use of EPO with renal fibrosis.

## EPO and Related Molecules

EPO is an acidic glycoprotein containing sialic acid whose precursor has 193 amino acids. After post-translational processing, the active protein has 165 amino acids and is highly glycosylated (19, 20). The serum EPO level in healthy individuals ranges from 15 to 25 U/L (21), and the oxygenation state of tissues regulates its production. EPO is a glycoprotein hormone secreted by the kidneys whose biological effects occur after it binds to its receptor (EPO-R), which occurs on the plasma membranes of target cells (22). EPO primarily targets hematopoietic cells in the bone marrow, which has high concentrations of EPO, and this is its main site of action (23). The binding of EPO to EPO-R on erythrocytes activates a variety of signaling pathways (24), thus stimulating cell proliferation, differentiation, and maturation (13). EPO can improve anemia by inducing erythropoiesis, thus indirectly ameliorating organ damages. Studies have shown that in predialysis patients with non-severe anemia, the early initiation of erythropoietin significantly slows the progression of kidney disease and delays the initiation of renal replacement therapy (25). Initial studies found EPO-R in erythroid progenitor cells, so researchers considered its function was limited to these cells, and gave little consideration to the role of EPO in the kidney and other tissues (26). More recent studies have shown expression of EPO-R by renal cells (including renal tubular cells, mesangial cells, and collecting duct cells), gastric epithelial cells, and some neurogenic cells (27, 28). Other studies have shown that EPO has cellular protective effects, through its interaction with the EPO-R, in the kidneys, brain, heart, and blood vessels, in that it regulates mitosis, reduces oxidative stress, inhibits apoptosis, promotes vascular repair, and has several other effects (24, 29). The clinical application of erythropoiesis stimulating agents (ESAs) was a milestone in the treatment of renal anemia, because these drugs greatly improve the prognosis of patients with chronic renal failure and provide important renoprotective effects (30–32).

However, due to the poor specificity and sensitivity of existing anti-EPO-R antibodies, there is still controversy about the function of EPO beyond its effect on erythropoiesis. Elliott et al. found that EPO-R protein was below the detection limit in tissues and renal cells and no evidence of EPO-R expression and function in the kidneys (33). A subsequent meta-analysis of clinical trials by Elliott et al. showed that ESAs had no clear renoprotective effect, at least in the included subjects (34). Thus, it is currently controversial whether functional EPO-R occurs on the plasma membranes of renal cells. Moreover, multiple clinical studies showed that ESAs provided no improvement in renal function after transplantation or acute kidney injury (35, 36). However, due to the poor specificity of the EPO-R assay and the discordance of *in vitro* and *in vivo* data in animal models, this topic needs further study.

## EPO and Renal Fibrosis

Renal fibrosis is a key feature of CKD and is the common pathologic manifestation and pathogenic outcome of end-stage renal disease (37). Renal interstitial fibroblast proliferation and the aberrant and persistent deposition of ECM are the main pathological features of renal fibrosis. This process begins when an inflammatory stimulus accelerates the transformation of epithelial cells and interstitial fibroblasts into myofibroblasts, which together produce excess ECM. Then, because of the decreased activity of matrix proteolytic enzymes and increased activity of protease inhibitors, excessive deposition of ECM occurs, leading to the formation of permanent fibrotic scars, thereby accelerating the progression of tubulointerstitial fibrosis (38, 39).

**Figure 1:**
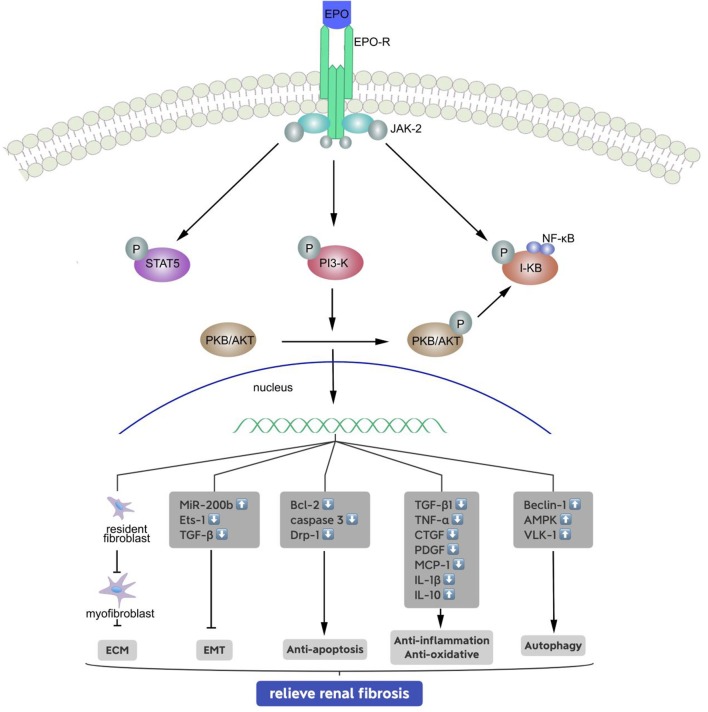
Signal transduction pathways of the erythropoietin receptor and mechanisms of relieving renal fibrosis. Binding of erythropoietin (EPO) causes conformational changes to the EPO receptor, phosphorylation of associated JAK-2, PI-3 kinase and I-kB molecules, and activation of signaling molecules and target genes: (1) inhibits the generation of stromal mesenchymal fibroblasts; (2) inhibits the EMT by upregulating miR-200b, and reducing of Ets-1 and TGF-β; (3) phosphorylates and inactivates proapoptotic molecules; (4) reduces inflammation by inhibiting the release of pro-inflammatory cytokines and anti-oxidative, and (5) enhances autophagy to some extent.

It is now known that EPO is a major multifunctional glycoprotein hormone that has protective functions in various organs and tissues. For example, an animal study demonstrated that EPO attenuated cardiac dysfunction by inhibiting interstitial fibrosis in diabetic rats (40). Another animal study reported the beneficial effects of EPO in diverse liver injuries, such as carbon tetrachloride-induced hepatic fibrosis (41). EPO also has renoprotective effects (Figure 1) (42, 43), and early treatment of anemia with EPO in CKD patients slows the development in renal dysfunction (44). Extensive research during recent years demonstrated that EPO can improve recovery from acute kidney injury (45). Administration of recombinant human EPO (rhEPO) reduces the production of urinary proteins and biomarkers associated with kidney injury and even with CKD. We therefore believe that EPO may also play a critical role in the development of renal fibrosis, but the mechanism of this effect requires further examination.

### EPO and Myofibroblasts

Myofibroblasts are fibroblasts containing actin, myosin, and other muscle-related proteins that provide these cells with contractile properties. Various stimuli and injuries can induce the activation and proliferation of renal interstitial fibroblasts, leading to the formation of active myofibroblasts. These renal myofibroblasts function as effector cells of the renal interstitial ECM, causing damage to the function of the kidney, and eventually leading to renal failure (46–48). Moreover, the population of novel myofibroblasts present in fibrotic kidneys can derive from renal tubular interstitial resident fibroblasts, bone marrow derived fibrocytes, vascular pericytes, the epithelial-mesenchymal transition (EMT), and the endothelial-mesenchymal transition (EndoMT) (49, 50).

There is an increasing body of evidence suggesting that interstitial myofibroblasts constitutively produce ECM, and that this leads to the development of glomerulosclerosis and tubulointerstitial fibrosis due to activation of TGF-β1 (51). The accumulation of matrix proteins, such as fibronectin and type I and III collagen, is a hallmark of renal fibrosis. Thus, under normal conditions, resident renal fibroblasts produce EPO in response to hypoxic insults to maintain physiological homeostasis. However, under pathologic conditions the resident renal fibroblasts transdifferentiate into myofibroblasts, which promote renal fibrosis by producing large amounts of extracellular matrix proteins rather than EPO (52). A study of mice showed that treatment with rhEPO significantly inhibited the accumulation of fibrocyte by inhibition of α-SMA upregulation, and thereby attenuating renal interstitial fibrosis (53). Another study of transgenic mice found that enhanced signaling mediated by hypoxia-inducible factor (HIF) in myofibroblast-transformed renal EPO-producing cells reactivated the synthesis of EPO, without influencing renal fibrosis or inflammation (54). However, studies of type 2 diabetic mice found that a continuous erythropoietin receptor activator (CERA) enhanced tissue repair by inhibiting the generation of stromal mesenchymal fibroblasts, and thus had a non-hematopoietic and tissue-protective role (55).

### EPO and the EMT

During the EMT, epithelial cells undergo a loss of cell-cell adhesion and apical-basal polarity. This is accompanied by increased expression of mesenchymal markers, rearrangement of the cytoskeleton, and increased cell dissociation, all of which contribute to development of the mesenchymal phenotype. The EMT is pathologically reactivated in, and contributes to, the progression of fibrosis (56). Tubulointerstitial fibroblasts, derived from tubular epithelial cells during the EMT, are among the most important effector cells facilitating the progression of renal fibrosis (57, 58). In the past few decades, many studies have examined the role of the EMT during organ fibrosis, wound healing, and cancer metastasis. These studies have demonstrated that TGF-β1 promotes renal fibrosis through the EMT by activation of Smad2/3 (59, 60). In support of this conclusion, animal studies have also shown that the renal EMT contributes to renal fibrosis. For example, Grande et al. concluded that the partial EMT drives renal fibrosis in mice (61). Therefore, it is important to identify interventions or drug therapies that could potentially reverse or inhibit the EMT in the kidney to improve clinical management of these patients.

Previous studies reported that EPO functions as an EMT inhibitor in the kidneys and was effective in ameliorating renal fibrosis (62). Imamura et al. reported that EPO inhibits tubulointerstitial fibrosis in remnant kidney by functioning as an inhibitor of the EMT (63). A recent study using HK-2 cells showed that administration of EPO markedly inhibited hypoxia-induced EMT by upregulating miR-200b expression via the repression of Ets-1. Another study of a unilateral ureteric obstruction (UUO) mouse model reported that rhEPO treatment reduced renal fibrosis by attenuating the EMT (64). Although the role of the EMT in renal fibrosis is not entirely clear, it is nonetheless important to identify therapeutic targets that may allow the prevention or reversal of the EMT and thereby slow the progression of CKD.

### EPO and Apoptosis

Apoptosis is the spontaneous and orderly death of cells in multicellular organisms that is regulated by specific genes to maintain the stability of the internal environment. In contrast to the passive process of necrosis, apoptosis is an active process in which there are specific changes in the expression of multiple genes. It is not a phenomenon of self-injury that occurs during pathological conditions, but a process that eliminates cells so that the organism is better adapted to its microenvironment. There are several signal transduction pathways associated with apoptosis; the major pathways are mitochondrial mediated apoptosis, endoplasmic reticulum mediated apoptosis, and death receptor-mediated apoptosis (65).

Renal fibrosis is accompanied by a significant reduction of parenchymal cells, including podocytes, mesangial cells, and tubular epithelial cells, and increased apoptosis also plays an important role. During renal interstitial fibrosis, there is evidence that apoptosis of renal tubular epithelial cells is closely related to renal tubular atrophy, and the increased apoptosis index during this process is an indication of the formation of extracellular matrix (66). Therefore, a targeted inhibition of apoptosis may be an effective strategy to delay or inhibit renal fibrosis.

EPO has many biochemical effects, in addition to its promotion of red blood cell production, and these include anti-inflammatory and anti-apoptotic effects. For example, EPO is the main regulator of erythroid progenitor cell proliferation and differentiation, and these are mediated via its anti-apoptotic effect (67). Many recent animal experiments have shown that EPO may delay the development of CKD and protect the kidneys by reducing the extent of interstitial fibrosis. For example, one study showed that the extent of renal fibrosis correlated positively with the number of apoptotic cells (68). Another study demonstrated that EPO provided a renoprotective and antiapoptotic effects by activation of ERK/p53 signaling (26). A study of renal interstitial fibrosis in a rat model reported that EPO had renoprotective effects due to its due to its down-regulation of dynamin-related protein-1 (Drp-1) (69), a protein with a major role in the mitochondrial mediated apoptosis pathway. A study by Nakazawa et al. showed that EPO inhibited the apoptosis of renal tubular cells, thereby reducing the extent of renal interstitial fibrosis (70). In the context of ischemia-reperfusion (IR) injury, reduced levels of pro-apoptotic genes in the Bcl-2 family can ameliorate the destruction of mitochondrial integrity and tubular cell apoptosis, and consequently inhibit renal injury (71, 72). Sharples et al. studied a rat kidney model of severe IR injury and demonstrated that EPO inhibited the apoptosis of proximal tubular epithelial cells and promoted significant cell proliferation at high doses, despite serum starvation (73).

### Anti-inflammatory Effects of EPO

Inflammation is a defensive response of living tissue that involves the vascular system and occurs following injury from factors such as infections, physical, chemical, or antigenic changes, or traumatic damage.

CKD is characterized by persistent renal inflammation that progresses to tubular interstitial fibrosis, renal failure, and end-stage renal disease (74). Elevated levels of inflammatory cytokines are associated with mortality, especially in patients with CKD. Various renal insults can activate tubulointerstitial cells and promote inflammatory cell infiltration, leading to the release of multiple vasoactive molecules and soluble cytokines that promote the progression of fibrosis. These molecules include TGF-β1, TNF-α, connective tissue growth factor, and platelet-derived growth factor. Secretion of these cytokines by tubulointerstitial and inflammatory cells alters the dynamic balance between synthesis and degradation of ECM proteins, eventually leading to the accumulation of ECM components and the development of fibrosis. Renal innate cells (mesangial cells, podocytes, and endothelial cells) also secrete a variety of fibrogenic cytokines such as TGF-β1 and TNF-α.

EPO also has an important non-haematopoietic effects in that it can reduce inflammation caused by injury, toxins, or hypoxia. Mateus et al. reported the anti-inflammatory effect of EPO was due to its inhibition of TNF-α and IL-1β production and stimulation of IL-10 production in an animal model of TNBS-induced colitis (75). Chang et al. found that subcutaneous injection of EPO into rats with UUO significantly reduced the expression of TNF-α and mononuclear cell chemotactic protein-1 (MCP-1), decreased inflammatory cell infiltration, inhibited interstitial fibrosis, and protected kidney function (76). Heme oxygenase-1 (HO-1) is an important antioxidant protein, Katavetin et al. demonstrated that EPO induces expression of HO-1, thereby reducing oxidative stress and delaying CKD progression (77).

### EPO and Autophagy

Autophagy is a conserved mechanism of cell self-degradation, in which lysosomes degrade damaged organelles and macromolecules so they can be reused. In general, autophagy is necessary to maintain cell homeostasis, and it has roles in slowing the progress of aging, promoting differentiation and development, increasing immunity and the clearance of microorganisms, and preventing the progression of tumors and other diseases (78, 79). However, excessive autophagy can have pathological effects. For example, Livingston et al., using pharmacological and genetic methods, demonstrated that sustained activation of autophagy in the proximal tubules promoted renal interstitial fibrosis in rats with UUO (80). In agreement, a study of an experimental model of neonatal necrotizing enterocolitis found that EPO reduced excessive autophagy, and contained cell damage (81). EPO also protects against rotenone-induced neurotoxicity in SH-SY5Y cells by enhancing autophagy-related signaling pathways (82). The renoprotective effects of ESAs in animal models may be due to their antiapoptotic effects. A study of kidney IR injury reported that ESAs had renoprotective effects by inducing autophagy (83). The antiapoptotic effects of EPO are dependent on JAK2 signaling and the phosphorylation of Akt by phosphatidylinositol 3-kinase. Increased Akt signaling is associated with suppression of cell apoptosis and promotion of renal fibrosis in IR injury-induced acute kidney injury (84). However, suppression of Akt phosphorylation accelerates tubular repair and inhibits renal fibrosis (85).

## Conclusion

The classical role of EPO is the regulation of red blood cell production, but many studies have reported that EPO also has many non-erythroid effects. These findings underline the importance of investigating the global actions of rhEPO and its derivatives in pre-clinical and clinical settings. Thus, EPO may be useful for protection of the kidney because of its effects on multiple pathways, in addition to its effect on red blood cell production. More specifically, EPO can prevent the development of renal failure during the end-stages of various renal diseases, and can actively control the fibrosis of renal interstitial tissue. However, the mechanism of EPO in the prevention and treatment of renal interstitial fibrosis is not fully understood. For this reason, additional studies are required to determine the optimal dose and timing of EPO administration and how to best avoid possible adverse reactions, such as thrombosis. The recent research and development of new types of EPO for treatment of anemia and other conditions may increase the applications of EPO for additional conditions. The results of this new research may also provide new insights into the mechanism by which rhEPO prevents interstitial fibrosis and slows the progression of CKD. Lastly, we believe that the many developments and unremitting efforts from multiple medical disciplines will eventually establish a basis for the use of EPO as a treatment for renal fibrosis and will help guide future research in this area.

## Author Contributions

YZ, XZhu, XH, and YD contributed conception and design of the study. XW and DZ organized the database. YZ wrote the first draft of the manuscript. LJ and XZha wrote sections of the manuscript. All authors contributed to manuscript revision, read and approved the submitted version.

### Conflict of Interest

The authors declare that the research was conducted in the absence of any commercial or financial relationships that could be construed as a potential conflict of interest.
